# Feasibility of a modified hybrid glubran-supported single-proglide technique for access closure during endovascular aneurysm repair

**DOI:** 10.3389/fcvm.2024.1426961

**Published:** 2024-07-04

**Authors:** Chen Xu, Guo-xiong Xu, Lei Chen, Zhi-xuan Zhang, Yi-qi Jin

**Affiliations:** Department of Vascular Surgery, The Affiliated Suzhou Hospital of Nanjing Medical University, Suzhou, China

**Keywords:** proglide devices, glubran glue, large bore access closure, endovascular aneurysm repair, access bleeding

## Abstract

**Objective:**

This study aimed to evaluate the feasibility of a hybrid Glubran-supported single-Proglide technique for large bore femoral access closure during percutaneous access endovascular aneurysm repair (EVAR).

**Methods:**

A retrospective cohort study was performed for all percutaneous EVARs at our center from January 2023 to June 2023. All patients received the hybrid Glubran-supported single-Proglide technique involving a mixture of surgical glue and Lipiodol injection after single suture placement for femoral access closure. Technical success was defined as achieving complete hemostasis without a bailout strategy. Vascular complications and bleeding were defined by Valve Academic Research Consortium-3 (VARC-3) criteria. Vascular access changes and 30-day mortality were recorded.

**Results:**

The technique success rate for the entire study population was 100% (55 femoral access in 37 patients; median age: 72; 78% males). The mean sheath size was 20.4 ± 2.3F. The mean manual compression time was 3.5 ± 1.4 min, the mean hemostasis time was 9.0 ± 2.5 min, and the mean procedural time was 103.9 ± 34.7 min. One patient (1.6%) developed an access site infection and recovered conservatively. No VARC-3 vascular complications and access changes were observed. No 30-day mortality happened.

**Conclusions:**

The hybrid Glubran-supported single-Proglide technique is feasible for large bore access closure during EVAR and may be a viable alternative; however, larger prospective studies are required to confirm its efficacy.

## Introduction

The number of patients undergoing percutaneous access endovascular aneurysm repair (EVAR) is steadily increasing with the benefit of improved patient comfort and reduced hospitalization (1–6) . Vascular access site management is still a major concern associated with significantly increased morbidity and mortality (7–9). The standard Perclose ProGlide (Abbott Vascular, Santa Clara, CA) technique for access closure has been widely adopted (10, 11). However, the use of double or, in some cases, triple ProGlide devices may have several drawbacks, including technical complexity, arterial stenosis, and an increased cost burden (9, 12).

Using a single ProGlide device plus Glubran glue (GEM Srl, Viareggio, Italy) at the arteriotomy site has recently been reported as an alternative strategy for large bore access closure (7). The cyanoacrylate-based Glubran glue is safe and effective for access hemostasis in peripheral arterial diagnostic and interventional angiography (13). Lipiodol (Guerbet, Pairs, French) is an oil-based solution designed for therapeutic interventions (14). Thus, a pre-implanted ProGlide followed by a mixture of Glubran glue and Lipiodol injection on the access site may aid in achieving large bore access hemostasis.

Our study aimed to evaluate the feasibility, efficacy, and safety of the hybrid Glubran-supported single-Proglide technique for access closure during EVAR.

## Materials and methods

### Study design

**Figure 1:**
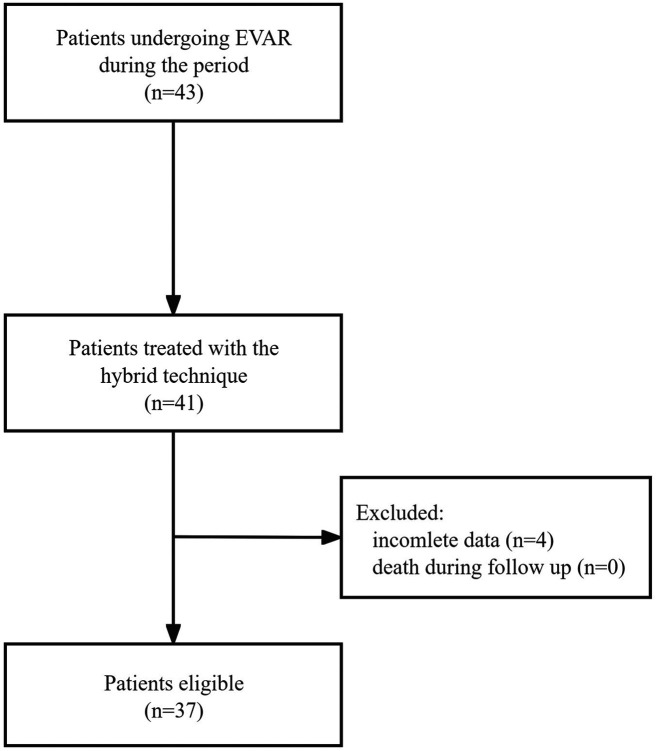
Study design.

This retrospective cohort study included all patients undergoing EVAR at our center from January 2023 to June 2023. Figure 1 illustrates the study design. Patients treated with a hybrid Glubran-supported single-Proglide technique for femoral access management during the study period were eligible for inclusion. Exclusion criteria included incomplete data, such as preoperative and postoperative imaging studies or death during follow-up. The institutional review board of our center approved the study, and all methods were performed in accordance with relevant guidelines and regulations (15).

### Vascular assessment

The evaluation and measurement of femoral vascular access were performed using computed tomography (CT) angiography, and ultrasonography (US) prospectively. Femoral artery depth was defined as the shortest distance in the puncture tract. Anterior wall artery calcification was considered to be present if calcification width was >2 mm at the puncture site. Access site calcification and tortuosity were defined as previously reported (0%–25% calcification of circumference—mild, 26%–75%—moderate, >75%—severe; 0–60 degrees of angulation—mild, 61–90 degrees—moderate, and >90 degrees—severe) (7). Two radiologists with over 5 years of experience measured changes in vascular size, including maximum and minimum artery diameter, using CT angiography and recorded the mean. A sonologist with over 8 years of experience measured changes in systolic peak velocity (SPV) of vascular access through US.

### Procedure

**Figure 2:**
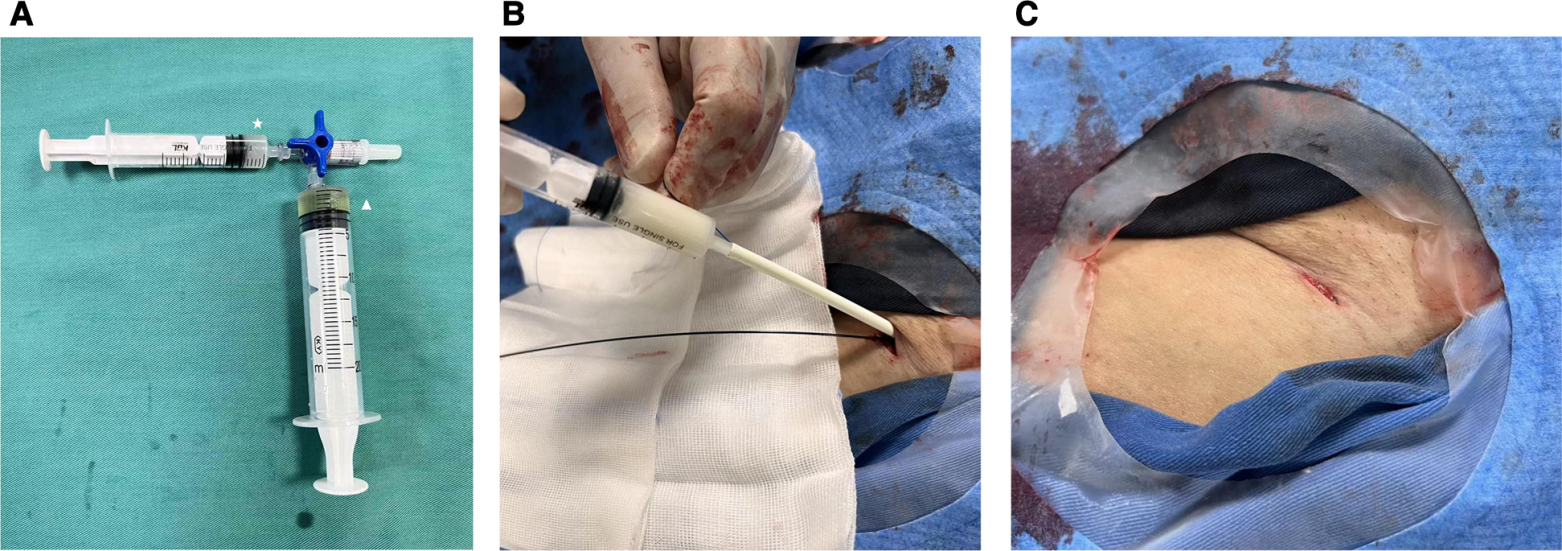
(**A**) A mixture of glubran glue and lipiodol in a ratio of 1:2 around the vessel wall and puncture route. Star: Glubran; triangle: Lipiodol. (**B**) The glue applicator (IntroducerII™, Terumo Medical, 10F) was advanced along the Proglide sutures and the injection was followed to achieve hemostasis. (**C**) Complete hemostasis of access site was achieved.

The current closure technique involved the standard deployment of a single ProGlide (at a 12 o'clock position) followed by the injection of a mixture of surgical glue (Glubran® 2) and Lipiodol (Lipiodol® Ultra Fluid) in a ratio of 1:2 around the vessel wall and puncture route (Figure 2A). Glubran glue, a cyanoacrylate-based tissue glue was proved safe and effective for access closure in a previous study (13). Notably, the specific Glubran applicator was not used in our study which was applied in previous studies in 5–6 or 7–8F sizes (7, 13). Instead, an improvised applicator was made by simply cutting off the valve from a regular 10F sheath (IntroducerII™, Terumo Medical) and use the remaining tube over the Perclose sutures (not over the guidewire) as the Glubran applicator (Figure 2B). Besides, Glubran was diluted with Lipiodol at a ratio of 1:2 in our study and it is particularly important to prevent the glue from entering the artery when injecting. We considered that a mixture of Glubran glue and Lipiodol could be a more effective closure, ensuring the glue is external to the arterial wall and preventing artery embolization under continuous fluoroscopy guidance.

General, lumbar, and local anesthesia were administered in the current study. The patients received 100 U/kg of heparin intravenously. An ultrasound-guided puncture confirmed a calcification-free vessel area for a guidewire advancement. A small skin incision was made at the access site, and the subcutaneous fat was dissected with mosquito forceps. The single Proglide was initially vertically inserted, and the EVAR procedure was performed according to standard practice.

**Figure 3:**
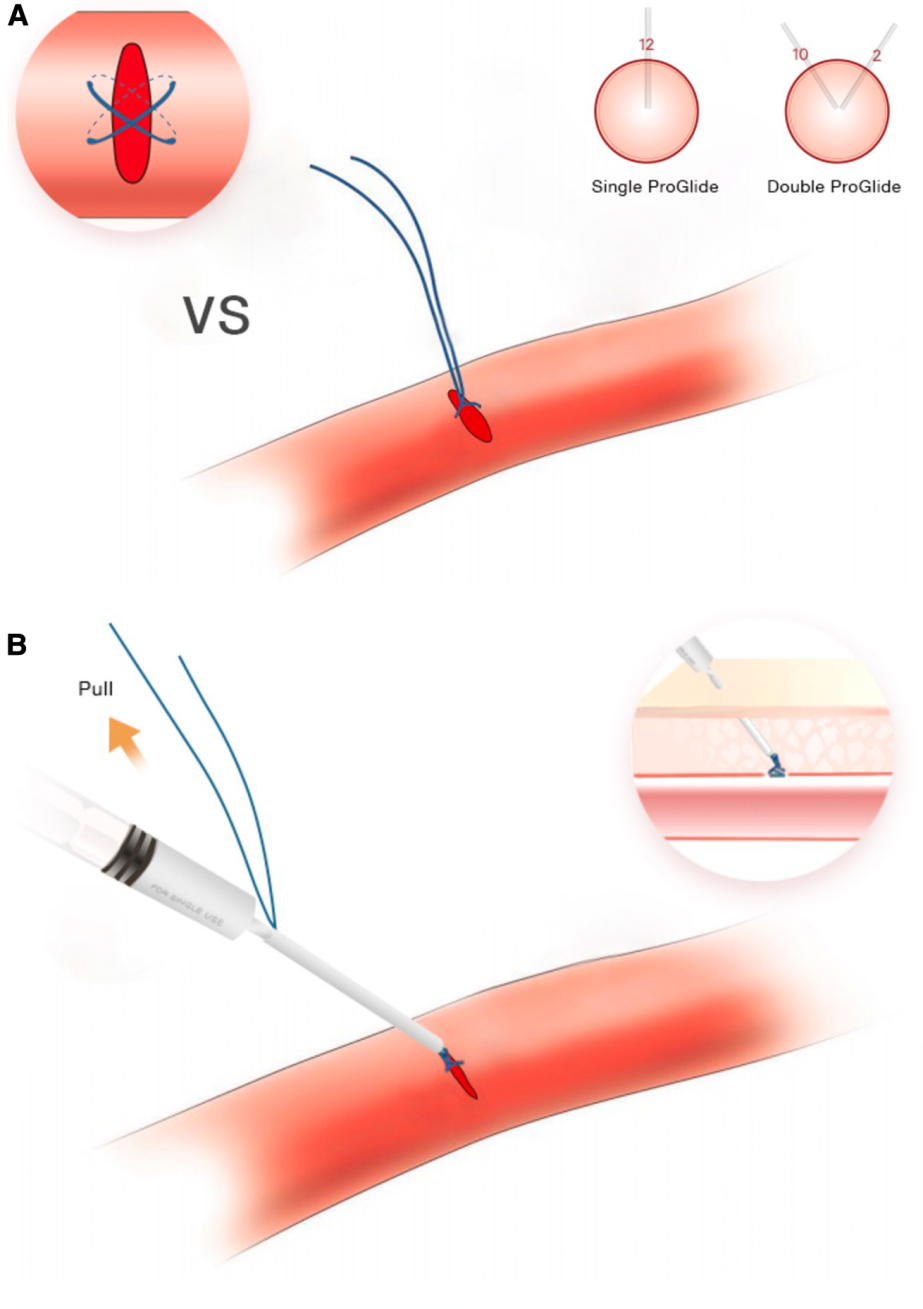
(**A**, **B**) The steps of the access closure.

After removing the large-caliber sheath, both Proglide sutures were meticulously tightened. If significant bleeding (persistent blooding under manual compressing) was observed, a second Proglide would be performed in the 12 or 2 o'clock position after the first one through a guidewire. Otherwise, the hybrid Glubran-supported single-Proglide technique was accepted as follows. While one operator manually compressed the proximal end of the access site, the glue applicator was advanced along the sutures until it made contact with the artery anterior wall. Once the surgeon felt the resistance of the knot and the angiography confirmed the extraluminal position, the fluoroscopy-guided injection was followed to achieve hemostasis. Finally, the glue applicator and guidewire were removed together, and a few additional manual compressions were required before hemostasis evaluation (Figure 2C). Access sites were evaluated immediately after the procedure and 24 h later by US to identify any access-related complications. The technique's steps are summarized in Figures 3.

Anticoagulation therapy is recommended for each EVAR, and antiplatelet therapy is used for fenestrated or branched EVAR. Gore Excluder and CTAG (W.L. Gore & Associates, Inc, Flagstaff, AZ, USA) and Ankura (Lifetech Scientific, Shenzhen, China) stent-graft systems with a sheath from 16 to 24 F were used.

### Endpoints

The study's primary endpoint was the closure technique's success, defined as achieving complete hemostasis without a bailout strategy including an unplanned endovascular or surgical intervention. Secondary endpoints included vascular complications and bleeding according to the Valve Academic Research Consortium-3 (VARC-3) criteria (16), manual compression time, hemostasis time, procedural time, length of hospital stay, need for unplanned intervention, additional use of Proglide, and 30-day mortality. Follow-up protocols, including CT angiography and US, were performed at 30 days to confirm complications and assess vascular access changes. The same surgeon obtained patient data from online clinical and standard operative records.

### Statistical analysis

Distributed data were expressed as mean ± standard deviation or interquartile range, and the *t*-test or Mann-Whitney *U*-test was used to compare the differences when appropriate. Categorical data are expressed as numbers and percentages, and Pearson's chi-square test or Fisher's exact test was utilized. A *P-value* of <0.05 was regarded as statistically significant. The SPSS software (version 19.0; SPSS Inc., Chicago, IL, USA) was used for Statistical analysis.

## Results

### Patients population

Baseline characteristics of patients were presented in Table 1. The current study enrolled 37 consecutive patients with a median age of 72 years and 55 femoral access sites. No patient was switched to a double Proglide technique. 83.8% of the patients had hypertension, 27.0% of the patients had diabetes, and 35.1% of the patients had cardiovascular disease. More than two-thirds of the patients received antiplatelet or anticoagulant therapy.

**Table 1:** Baseline characteristics (patients, *n* = 37).

Age, years	68.1 ± 12.2
Sex, male	29 (78.4)
Body mass index, kg/m^2^	25.1 ± 2.8
Hypertension	31 (83.8)
Diabetes mellitus	10 (27.0)
Cardiovascular disease	15 (35.1)
Chronic kidney disease	2 (5.4)
Peripheral arterial disease	8 (21.6)
Dyslipidemia	18 (48.6)
Diagnosis	
Thoracic aortic aneurysm	1 (2.7)
Abdominal aortic aneurysm	13 (35.1)
Aortic dissection	21 (56.8)
Penetrating aortic ulcer	2 (5.4)
Antiplatelet therapy	14 (37.8)
Anticoagulation therapy	17 (45.9)
Antiplatelet + anticoagulation therapy	7 (18.9)
Current smoking	11 (29.7)
Previous open cut-down	1 (2.7)
Previous use of vascular closure device	5 (13.5)

### Procedure details

We included standard (48.6%) and fenestrated or branched (51.4%) EVAR procedures with sheaths averaging 20.4 ± 2.3 F. Table 2 depicts various access-site anatomy. Severe calcification was found in 10.9% of the patients. Extremely tortuous vessels were present in 5.5% of the patients. Table 3 summarizes the procedure details. The mean manual compression time was 3.5 ± 1.4 min, and the mean procedural time was 103.9 ± 34.7 min. The mean time to complete hemostasis was 9.0 ± 2.5 min, and the mean time to discharge from the hospital was 5.3 ± 2.9 days.

**Table 2:** Access site anatomy (patients, *n* = 37; groins, *n* = 55).

Unilateral femoral access	18 (48.6)
Common iliac artery-minimum diameter, mm	10.2 ± 1.2
External iliac artery-minimum diameter, mm	9.1 ± 1.1
Common femoral artery-minimum diameter, mm	7.9 ± 1.1
Femoral artery depth, cm	3.3 ± 1.2
Anterior wall artery calcification	5 (9.1)
Access site calcification	
Mild/moderate	49 (89.1)
Severe	6 (10.9)
Access artery tortuosity	
Mild/moderate	52 (94.5)
Severe	3 (5.5)

**Table 3:** Procedure characteristics (patients, *n* = 37; groins, *n* = 55).

Procedure priority	
Elective	23 (62.2)
Emergency	14 (37.8)
Procedure type	
EVAR	15 (40.5)
TEVAR	3 (8.1)
F/BEVAR	19 (51.4)
Anesthesia	
Local	5 (13.5)
Lumbar	13 (35.1)
General	19 (51.4)
Main graft type	
Ankura	26 (70.3)
Excluder	11 (29.7)
Sheath size, Fr	
Right	21.6 ± 1.7
Left	18.0 ± 1.4
Procedural time, min	103.9 ± 34.7
Manual compression time, min	3.5 ± 1.4
Time to complete hemostasis, min	9.0 ± 2.5
Time to discharge, days	7.3 ± 2.9

### Endpoints and vascular access changes

The Proglide plus Glubran technique had a success rate of 100.0%. Only one patient (1.6%) developed an access site infection and managed conservatively with antibiotics. There were no VARC-3 vascular complications, and no additional Proglide insertions were required. No patients received unplanned endovascular or surgical intervention for vascular access complications. There was no in-hospital or 30-day mortality (Table 4). Vascular access changes in maximum artery diameter were 8.84 ± 1.02 vs. 8.79 ± 0.98 mm, respectively (*p* = 0.834). Changes in minimum artery diameter were 7.92 ± 1.10 vs. 7.65 ± 1.31 mm, respectively (*p* = 0.354). Changes in SPV were 77.82 ± 12.21 vs. 78.61 ± 12.55 cm/s, respectively (*p* = 0.783). There was no evidence of vascular stenosis overall, and this technique did not increase the risk of vessel narrowing (Table 5).

**Table 4:** Endpoints (groins, *n* = 55).

Primary endpoint	
Technique success	55 (100)
Access-related complications	
Minor VARC-3 bleeding	0
Major VARC-3 bleeding	0
Infection	1 (1.6)
Pseudoaneurysm	0
Occlusion/stenosis	0
Additional use of Proglide	0
Unplanned endovascular/surgical intervention	0
30-day mortality	0

**Table 5:** Access site changes (groins, *n* = 55).

	Before	30 days	*p*-value
Maximum artery diameter, mm	8.84 ± 1.02	8.79 ± 0.98	0.834
Minimum artery diameter, mm	7.92 ± 1.10	7.65 ± 1.31	0.354
Systolic peak velocity, cm/s	77.82 ± 12.21	78.61 ± 12.55	0.783

## Discussion

According to the findings of this single-center study, the hybrid Glubran-supported single-Proglide technique is a feasible and safe strategy for large bore access closure after EVAR. The technical success rate was 100%, with no glue artery embolization cases. No VARC-3 vascular complications were experienced, and no patients received unplanned intervention. The overall complication rate was 1.6%, consistent with previous studies involving double ProGlide devices of 0%–11.4% (5, 9, 17–19). However, considering the small cohort in our study, larger prospective studies are required to confirm our findings.

A double ProGlide technique for access closure after interventional procedures has proved safe and effective in previous studies (10, 11). Therefore, most EVAR centers use two ProGlide devices to preclose the access site. However, this technique has limitations for patients with peripheral vascular disease, vessel calcification, and obesity. Shoeib (19) et al. reported that the double-suturing devices reduce the minimum vessel diameter by an average of 1 mm in TAVI patients. Multiple studies validated a higher rate of arterial strictures in patients treated with double Proglide techniques (9, 17). Smith (20) et al. indicated that a second Proglide may increase the risk of suture fracture during sheath exchange and lead to poor access site closure.

It has recently been suggested that using a single ProGlide device for access closure as a strategy may reduce the overall procedure duration and minimize vascular complications when compared to double ProGlide devices in patients undergoing TAVI (21). However, the current evidence is still insufficient, particularly for EVAR patients. Hemostasis may not be completely achieved using a single ProGlide device based on clinical experience.

Therefore, we considered a single ProGlide placement followed by a mixture of Glubran and Lipiodol injection to achieve optimal access site hemostatic control under continuous fluoroscopy guidance. The hybrid technique achieves hemostasis at two distinct action levels; the vessel wall level (suture) and the subcutaneous tissue level (glue/Lipiodol). The Glubran glue has been proven safe and effective for access hemostasis (5–8 F) in the previous study (13). Sorropago (7) et al. reported using a combination of Proglide and Glubran glue as an alternative strategy to achieve hemostasis at large bore access sites (16–24 F). In our study, we used a combination of Glubran and Lipiodol in a ratio of 1:2. This may be more effective for closure, ensuring that the glue is external to the arterial wall and reduces the risk of artery embolization under continuous fluoroscopy guidance. Notably, it is important to emphasize the achievement of 100% closure without the need for an extra Proglide using this hybrid technique, and the additional advantage was the effective hemostasis achieved in a vessel with severe calcification and extreme tortuosity.

Moreover, it has been suggested that a hybrid strategy combining suture and plug vascular closure devices may have greater efficacy and a lower risk of subsequent peripheral ischemia after TAVI than double suture devices (9, 22). Gmeiner (17) et al. demonstrated that this strategy reduces arterial wall constriction and tension while retaining the benefits of both devices. For Angioseal, the device requires the operator to lose wire access during deployment. Once it fails to achieve enough hemostasis, options become limited to manual pressure or unplanned intervention. For Manta, early feasibility trials and retrospective analyses reported promising results, however, a higher rate of access-related vascular complications compared with the Dual Proglide strategy was displayed in recent studies (23, 24). To further evaluate the safety and efficacy of access hemostasis, it is necessary to conduct large-scale comparative studies between plug devices and Glubran glues.

In the current study, one patient (1.6%) developed an access site infection and recovered conservatively before discharge. Based on our limited experience, wound infection may occur if the glue/Lipiodol is injected excessively to pursue complete hemostasis. Therefore, a 1–2 ml mixture injection is recommended sufficient. The ratio of Glubran and Lipiodol was 1:2 in this study. Our decision was based on our shared clinical experience. Additional research should be conducted to confirm the optimal amount and proportion. Moreover, it is crucial to ensure the standard deployment of the first single ProGlide (at a 12 o'clock position), and all patients must undergo a complete fluoroscopy-guided injection to prevent artery embolization. Finally, maintain guidewire access until complete hemostasis is confirmed to permit further therapy.

Our research has a few limitations. The study's small sample size and retrospective nature may have affected data reliability and clinical outcomes, especially the mean BMI of the study cohort is low which suggest quite slim patients. More studies with a larger sample size should therefore be conducted to further evaluate the safety and efficacy of this hybrid strategy, especially compared to the current double ProGlide technique. Besides, although no case was occurred in our study, the risk of glue embolization still exist, more studies are needed to demonstrate the safety of this technique.

## Conclusion

The hybrid Glubran-supported single-Proglide technique is a feasible method for large bore access closure up to 24 F during EVAR, with a high technique success rate and no VARC-3 vascular complications. As a result, this strategy may be a viable alternative for clinical therapy but should be supported by larger prospective studies.

## Data Availability

The raw data supporting the conclusions of this article will be made available by the authors, without undue reservation.
